# Supplementary material for: Kinetics of Isothermal Dumbbell Exponential Amplification: Effects of Mix Composition on LAMP and Its Derivatives
Source: Biosensors (Basel). 2022 May 18;12(5):346. doi: 10.3390/bios12050346 (PMC9138685; doi:10.3390/bios12050346)
Supplement: Supplementary file 1 [file biosensors-12-00346-s001.zip › biosensors-1686967-supplementary.pdf]

Supplementary Materials: Kinetics of Isothermal Dumbbell Exponential Amplification: Effects of Mix Composition on LAMP and its Derivatives

Maud Savonnet [1,2], Mathilde Aubret [1,2], Patricia Laurent[2], Yoann Roupioz[1], Myriam Cubizolles[2,*] and Arnaud Buhot [1,*]

1. Standard LAMP reaction

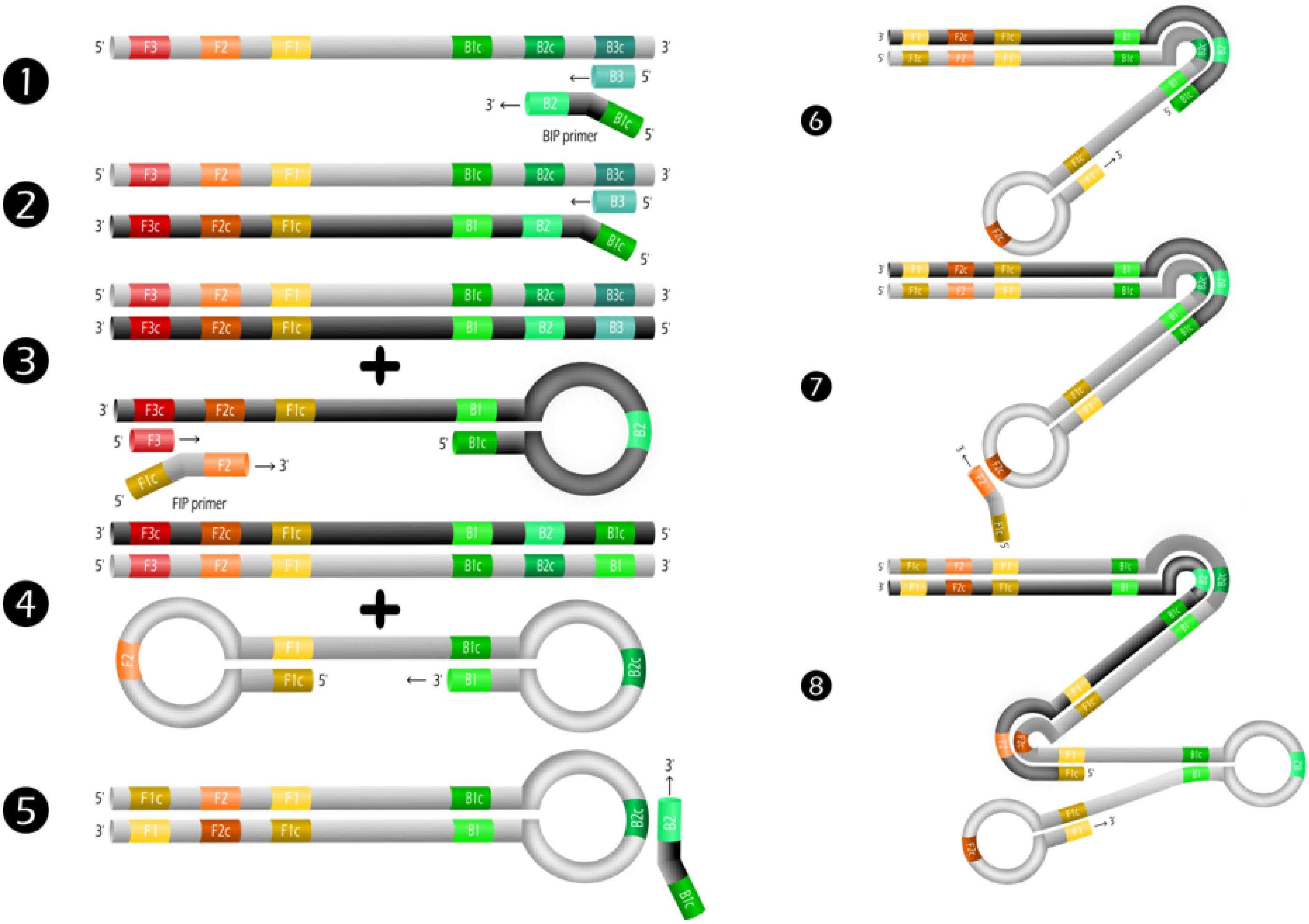

Figure S1. Standard LAMP reaction with four primers (B3, F3, BIP and FIP). Steps 1 to 4 depict the first stage of LAMP reaction. This first stage consists in the formation of a dumbbell from a generic target thanks to the four primers. The stage 2 requires only the two primers BIP and FIP for the isothermal dumbbell exponential amplification.

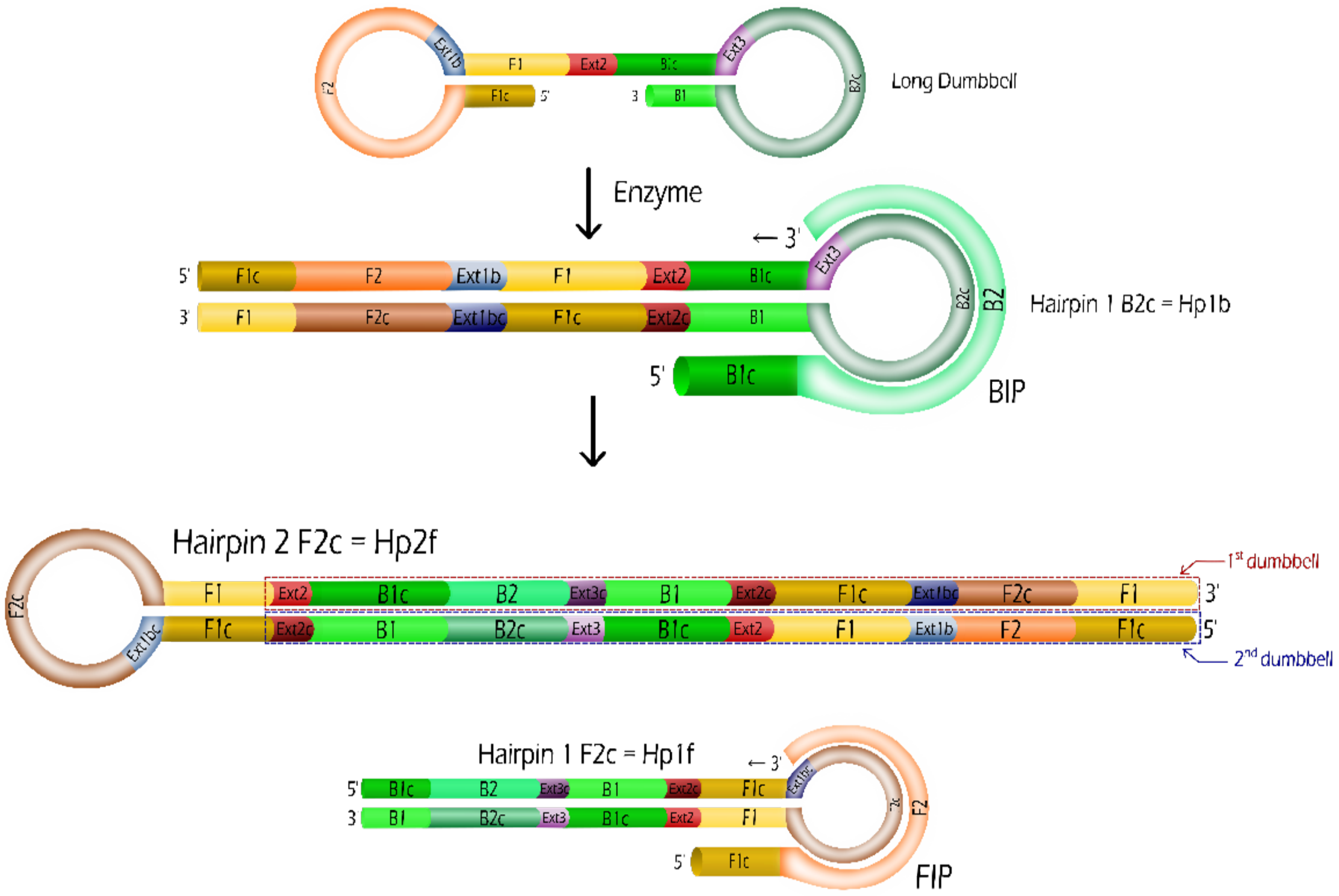

Figure S2. Incorporation of extensions for the dumbbell LD. Formation of hairpin Hp1b from the dumbbell LD followed by the duplication of hairpins: Two hairpins Hp1f and Hp2f are created from the hairpin Hp1b with the use of a single BIP and enzymes. All the three extensions are incorporated inside Hp2f.

2. Primer limiting kinetic rate

In this section, the hybridization of FIP or BIP primers are the limiting kinetic steps. The hybridization are supposed to be similar for both reactions and mainly depend on the rate of hybridization k_{hyb} of the primer (either BIP or FIP) to the hairpin. The kinetics rules are :

$$\frac{d}{dt}c(0,t) = 0 \tag{1}$$

$$\frac{d}{dt}c(n,t) = k_{hyb}c_p(t)c(n-1,t) \qquad \text{for } n \geq 1 \tag{2}$$

leading to

$$\frac{d}{dt}c_{Hp}(t) = k_{hyb}c_p(t)\sum_{n=1}^{\infty} c(n-1,t) = k_{hyb}c_p(t)c_{Hp}(t) \tag{3}$$

$$\frac{d}{dt}l_{Hp}(t) = 2l_D k_{hyb}c_p(t)\sum_{n=1}^{\infty} 2^{n-1}c(n-1,t) + l_D k_{hyb}c_p(t)c_{Hp}(t)/2 \tag{4}$$

$$\frac{d}{dt}l_{Hp}(t) = k_{hyb}c_p(t)(2l_{Hp}(t) - l_D c_{Hp}(t)/2) \tag{5}$$

The length grows more rapidly than the concentration of strands due to the exponential increase of the stem length of the hairpins produced. Indeed, if we assume that the rate constant k_{hyb} does not depend on n, the differential equations for $c_{Hp}(t)$ and $l_{Hp}(t)$ may be combined to lead:

$$\frac{dc_{Hp}}{dl_{Hp}} = \frac{c_{Hp}}{2l_{Hp} - l_D c_{Hp}/2} \tag{6}$$

$$(2l_{Hp} - l_D c_{Hp}/2)dc_{Hp} = c_{Hp}dl_{Hp} \tag{7}$$

By introducing, $l_{hp} = ac_{Hp}^2 + bl_D c_{Hp}$, we obtain:

$$(2ac_{Hp}^2 + 2bl_D c_{Hp} - l_D c_{Hp}/2)dc_{Hp} = c_{Hp}(2ac_{Hp} + bl_D)dc_{Hp} \tag{8}$$

$$(bl_D - l_D/2)c_{Hp}dc_{Hp} = 0 \tag{9}$$

Thus, $b = 1/2$ is a solution and $a = l_D/c_D$ is determined from the initial condition: $l_{Hp0} = 3l_D c_D/2 = ac_D^2 + l_D c_D/2$ where c_D is the initial concentration of dumbbells. We deduce the general expression for l_{Hp} as function of c_{Hp}:

$$l_{Hp}(t) = l_D\left(\frac{c_{Hp}^2(t)}{c_D} + \frac{c_{Hp}(t)}{2}\right) \tag{10}$$

This equation is valid as soon as the rate constant k_{hyb} is hairpin length independent.

2.1. Constant primer concentration

We assume at least for short times that the concentration of primers is constant: $c_p(t) = c_P$. Then, the concentration of hairpins follows:

$$\frac{d}{dt}c_{Hp}(t) = k_{hyb}c_P c_{Hp}(t) \tag{11}$$

$$c_{Hp}(t) = c_D \exp(k_{hyb}c_P t) \tag{12}$$

For the length of hairpins,

$$l_{Hp}(t) = l_D \left(\frac{c^2_{Hp}(t)}{c_D} + \frac{c_{Hp}(t)}{2} \right) \quad (13)$$

$$l_{Hp}(t) = l_D c_D \Big(\exp(2k_{hyb} c_P t) + \exp(k_{hyb} c_P t)/2 \Big). \quad (14)$$

Both the concentration and length of hairpins are exponentially increasing as expected with a rate two times larger for the length than for the concentration of hairpins.

2.2. Saturation due to finite primer concentration

Since the number of initial primers is finite, at long timescales, the hairpin concentration may saturate due to a lack of primers. Indeed, for each new hairpin produced a primer disappears. So both concentrations of hairpins and primers are related : $c_{Hp}(t) + c_p(t) = c_D + c_P = c_{tot}$. Thus,

$$\frac{d}{dt} c_{Hp}(t) = k_{hyb}(c_{tot} - c_{Hp}(t)) c_{Hp}(t) \quad (15)$$

$$k_{hyb} t = \int_{c_D}^{c_{Hp}(t)} \frac{dc}{c(c_{tot} - c)}$$

$$k_{hyb} c_{tot} t = \int_{c_D}^{c_{Hp}(t)} \frac{(c_{tot} - c) + c}{c(c_{tot} - c)} dc$$

$$k_{hyb} c_{tot} t = \int_{c_D}^{c_{Hp}(t)} \frac{dc}{c} + \int_{c_D}^{c_{Hp}(t)} \frac{dc}{c_{tot} - c}$$

$$k_{hyb} c_{tot} t = \ln \frac{c_{Hp}(t)}{c_D} + \ln \frac{c_{tot} - c_D}{c_{tot} - c_{Hp}(t)}$$

$$\exp(k_{hyb} c_{tot} t) = \frac{(c_{tot} - c_D) c_{Hp}(t)}{(c_{tot} - c_{Hp}(t)) c_D}$$

$$\exp(k_{hyb} c_{tot} t)(c_{tot} - c_{Hp}(t)) c_D = c_P c_{Hp}(t)$$

$$c_{Hp}(t) = \frac{c_{tot} c_D \exp(k_{hyb} c_{tot} t)}{c_P + c_D \exp(k_{hyb} c_{tot} t)} \quad (16)$$

With those assumptions, the concentration of hairpins follows a logistic function as generally fitted for LAMP experiments. The primer concentration decreases exponentially:

$$c_p(t) = c_{tot} - c_{Hp}(t) = \frac{c_{tot} c_P}{c_P + c_D \exp(k_{hyb} c_{tot} t)} \quad (17)$$

As shown previously,

$$l_{Hp}(t) = l_D \left(\frac{c^2_{Hp}(t)}{c_D} + \frac{c_{Hp}(t)}{2} \right) \quad (18)$$

$$l_{Hp}(t) = \frac{l_D}{c_D} \left(\frac{c_{tot} c_D \exp(k_{hyb} c_{tot} t)}{c_P + c_D \exp(k_{hyb} c_{tot} t)} \right)^2 + \frac{l_D}{2} \left(\frac{c_{tot} c_D \exp(k_{hyb} c_{tot} t)}{c_P + c_D \exp(k_{hyb} c_{tot} t)} \right)$$

$$l_{Hp}(t) = \frac{l_D c_{tot} c_D}{2} \frac{2c_{tot} \exp(2k_{hyb} c_{tot} t) + \exp(k_{hyb} c_{tot} t)(c_P + c_D \exp(k_{hyb} c_{tot} t))}{(c_P + c_D \exp(k_{hyb} c_{tot} t))^2}$$

$$l_{Hp}(t) = \frac{l_D c_{tot} c_D}{2} \frac{(2c_{tot} + c_D) \exp(2k_{hyb} c_{tot} t) + c_{tot} c_P \exp(k_{hyb} c_{tot} t)}{(c_P + c_D \exp(k_{hyb} c_{tot} t))^2} \quad (19)$$

3. Saturation due to finite dNTPs concentration

Contrarily to the primer concentration, the dNTPs concentration $c_n(t)$ couples with the length of the hairpins $l_{Hp}(t)$ instead of their concentration $c_{Hp}(t)$. Thus, the set of coupled equations is the following:

$$\frac{d}{dt}c_{Hp}(t) = \frac{k_E c_n(t)}{c_n(t)+K_D}c_{Hp}(t) \tag{20}$$

$$\frac{d}{dt}l_{Hp}(t) = \frac{k_E c_n(t)}{c_n(t)+K_D}(2l_{Hp}(t)-l_D c_{Hp}(t)/2) \tag{21}$$

$$c_n(t) = l_{tot}-l_{Hp}(t) \tag{22}$$

with $l_{tot} = c_{n0} + l_{Hp}(0)$. For simplicity, we may neglect $l_D c_{Hp}(t)$ compared to $l_{Hp}(t)$,

$$\frac{d}{dt}l_{Hp}(t) = \frac{2k_E(l_{tot}-l_{Hp}(t))l_{Hp}(t)}{l_{tot}-l_{Hp}(t)+K_D} \tag{23}$$

$$dt = \frac{l_{tot}-l_{Hp}+K_D}{2k_E(l_{tot}-l_{Hp})l_{Hp}}dl_{Hp} \tag{24}$$

A simple integration on the timescale $[t_{co}, t]$ leads to:

$$2k_E(t-t_{co}) = \int_{l_{Hp}(t_{co})}^{l_{Hp}(t)} \frac{l_{tot}-l+K_D}{(l_{tot}-l)l}dl \tag{25}$$

$$= \int_{l_{Hp}(t_{co})}^{l_{Hp}(t)} \frac{1}{l}dl + \int_{l_{Hp0}}^{l_{Hp}} \frac{K_D}{(l_{tot}-l)l}dl \tag{26}$$

$$= \ln\frac{l_{Hp}(t)}{l_{Hp}(t_{co})} + \frac{K_D}{l_{tot}}\int_{l_{Hp}(t_{co})}^{l_{Hp}(t)} \frac{(l_{tot}-l)+l}{(l_{tot}-l)l}dl \tag{27}$$

$$= \ln\frac{l_{Hp}(t)}{l_{Hp}(t_{co})} + \frac{K_D}{l_{tot}}\left(\ln\frac{l_{Hp}(t)}{l_{Hp}(t_{co})} + \ln\frac{l_{tot}-l_{Hp}(t_{co})}{l_{tot}-l_{Hp}(t)}\right) \tag{28}$$

$$\exp(2k_E(t-t_{co})) = \left(\frac{l_{Hp}(t)}{l_{Hp}(t_{co})}\right)^{\frac{l_{tot}+K_D}{l_{tot}}}\left(\frac{l_{tot}-l_{Hp}(t_{co})}{l_{tot}-l_{Hp}(t)}\right)^{\frac{K_D}{l_{tot}}} \tag{29}$$

The expression is no more a logistic function.

4. Figures from Experimental results

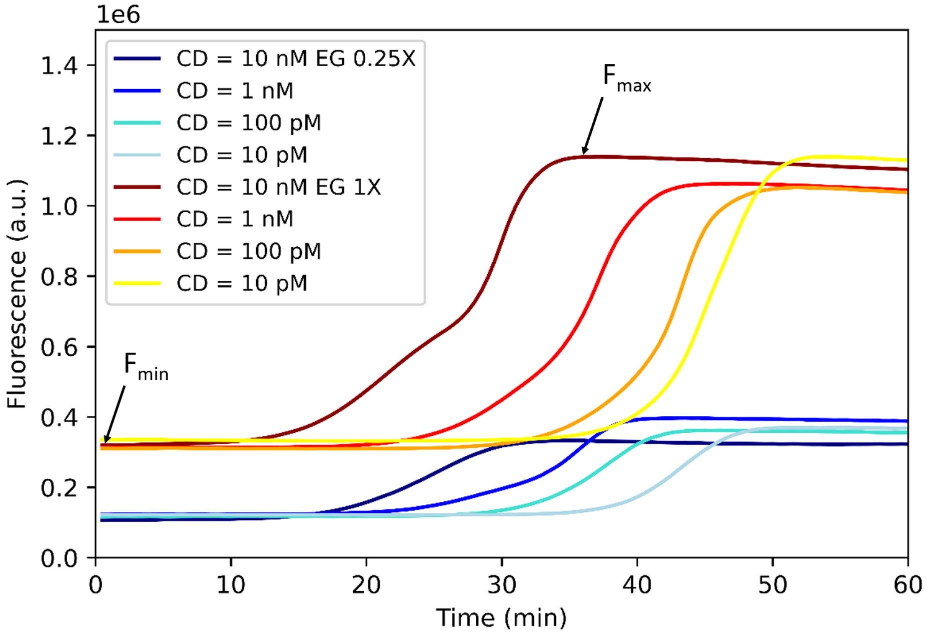

Figure S3. EvaGreen concentration effects on fluorescence measurements. LAMP was performed with MD dumbbell at 4 concentrations ($10^4, 10^3, 10^2$, 10 pM respectively) and two different concentrations of EvaGreen dyes (1X and 0.25X). As an illustration, the minimum and maximum fluorescent levels considered for the normalization has been depicted on the brown curve.

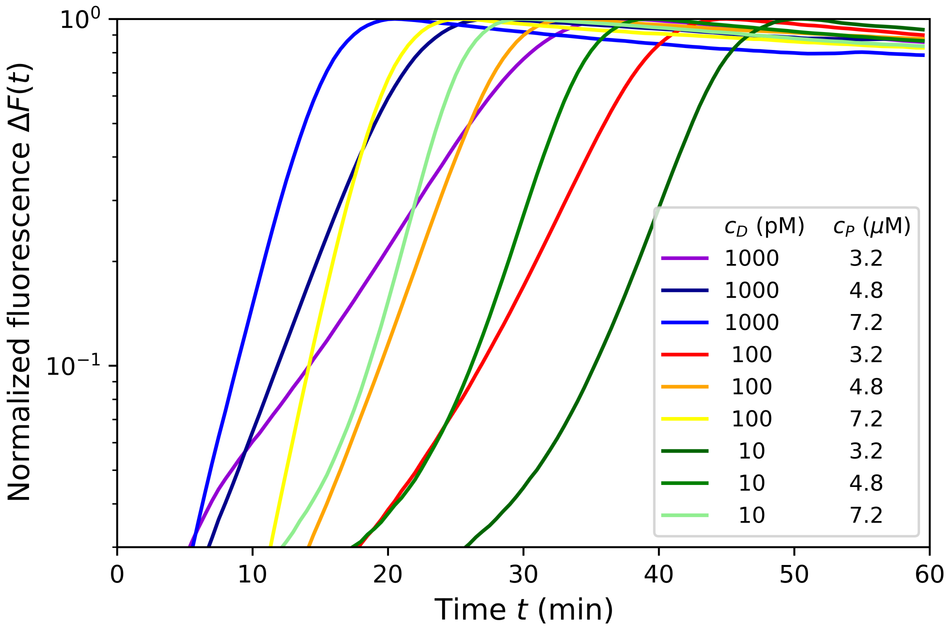

Figure S4. Normalized fluorescence ΔF in logarithmic scale on the y-axis as function of time for three concentration of primers ($c_P = 3.2, 4.8, 7.2$ μM) and three dumbbell concentrations $c_D = 1000, 100, 10$ pM.

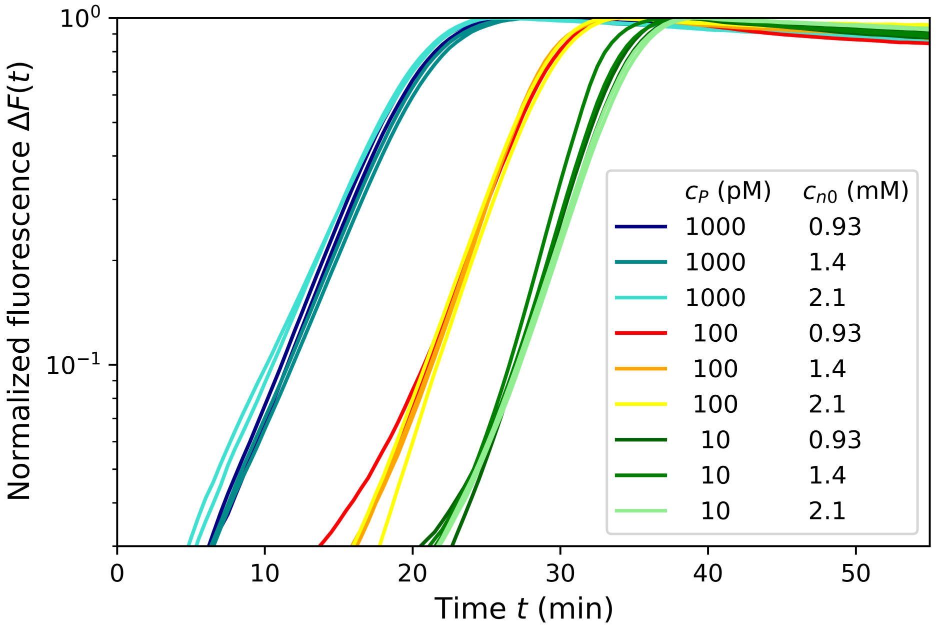

Figure S5. Normalized fluorescence ΔF as function of time in logarithmic scale for various concentrations of dNTPs (respectively $c_{n0} = 0.93, 1.4, 2.1$ mM) illustrates the lack of dependence on c_{n0} for three dumbbell concentrations $c_D = 1000, 100, 10$ pM.
